# Variability of soybean response to rhizobia inoculant, vermicompost, and a legume-specific fertilizer blend in Siaya County of Kenya

**DOI:** 10.1016/j.still.2019.06.007

**Published:** 2019-11

**Authors:** Catherine Mathenge, Moses Thuita, Cargele Masso, Joseph Gweyi-Onyango, Bernard Vanlauwe

**Affiliations:** a Department of Agricultural Science and Technology, Kenyatta University, P.O BOX 43844-00100, Nairobi, Kenya; b International Institute of Tropical Agriculture, c/o ICIPE, Duduville, Kasarani, P.O. Box 30772-00100, Nairobi, Kenya; c International institute of Tropical Agriculture, Yaounde, Cameroon

**Keywords:** Rhizobia inoculation, Nodule occupancy, Nodule effectiveness, Vermicompost, Grain yield

## Abstract

Rhizobia inoculation can increase soybean yield, but its performance is influenced by among others soybean genotype, rhizobia strains, environment, and crop management. The objective of the study was to assess soybean response to rhizobia inoculation when grown in soils amended with urea or vermicompost to improve nitrogen levels. Two greenhouse experiments and one field trial at two sites were carried out. The first greenhouse experiment included soils from sixty locations, sampled from smallholder farms in Western Kenya. The second greenhouse experiment consisted of one soil selected among soils used in the first experiment where inoculation response was poor. The soil was amended with vermicompost or urea. In the two greenhouse experiments, Legumefix (inoculant) + Sympal (legume fertilizer blend) were used as a standard package. Results from the second greenhouse experiment were then validated in the field. Analysis of variance was done using SAS statistical software and mean separation was done using standard error of the difference for shoot biomass, grain yield nodulation, nodule effectiveness and nutrient uptake. In the first greenhouse trial, soybean response to inoculation was significantly affected by soil fertility based on nodule fresh weight and shoot biomass. Soils with low nitrogen had low to no response to inoculation. After amendment, nodule fresh weight, nodule effectiveness, nodule occupancy, and shoot dry biomass were greater in the treatment amended with vermicompost than those amended with urea (Legumefix + Sympal + vermicompost and Legumefix + Sympal + urea) respectively. Under field conditions, trends were similar to the second experiment for nodulation, nodule occupancy and nitrogen uptake resulting in significantly greater grain yields (475, 709, 856, 880, 966 kg ha^−1^) after application of vermicompost at 0, 37, 74, 111, and 148 kg N ha^−1^ respectively. It was concluded that soybean nodulation and biological nitrogen fixation in low fertility soils would not be suppressed by organic amendments like vermicompost up to 148 kg N ha^−1^.

## Highlights

• Low response to Rhizobia inoculation observed in soil with low available C and N.
• Legume fertilizer blend did not enhance nodulation in soil of very low available N.
• Vermicompost amendment gave higher nodulation, nodule occupancy, nitrogen uptake.
• Vermicompost yield higher biomass & grain yield in greenhouse and field trials.

## 1 Introduction

Soybean (*Glycine max* L. Merr) is one of the world’s most important legumes in terms of production and trade and has been a dominant oilseed since the 1960s (Smith and Huyser, 1987). The crop is well known for its high protein content (about 40%) among the most cultivated crops (Li-Juan and Ru-Zhen, 2010). Additionally, it can improve soil properties and soil biological health by soil nitrogen enrichment through biomass addition and N_2_ fixation (Singh and Shivakumar, 2010). In sub-Saharan Africa (SSA), where over 80% of the soil are nitrogen deficient (Liu et al., 2010), and over 39% of the children under 5 years are malnourined and stunted. This has been related to deficiency of essential nutrients in most of diets, particularly proteins (Müller and Krawinkel, 2005), which has contributed to over one third of child deaths (Bain et al., 2013). Integration of soybean in the smallholder farming systems would thus not only improve human nutrition when the crop is included in the diet practices but also soil productivity. Such benefits would however, materialize when good agronomic practices, including integrated soil fertility management, are implemented in soybean production systems.

Crop production, including soybean, faces many constraints which includes among others abiotic and socio-economic factors accounting for production discrepancies across regions in sub-Saharan Africa (SSA). Therefore, grain yields remain low compared to other regions in the world (Mpepereki et al., 2000). Integrated soil fertility management (ISFM), has been proposed as a viable way towards the sustainable intensification of smallholder agriculture (Vanlauwe et al., 2010). The high cost of inputs for nutrient replenishment or soil amendment has, however, limited their adoption by resource-constrained smallholder farmers (Yakubu et al., 2010). Utilization of soybean varieties with high biological nitrogen fixation potential and application of rhizobia inoculants would represent cost-effective option to reduce mineral N application (Kueneman et al., 1984; Musiyiwa et al., 2005; Zengeni and Giller, 2007; Bekere and Hailemariam, 2012; Thuita et al., 2012; Ronner et al., 2016). Studies on N_2_ fixation in soybean using different methodologies revealed that soybean shows a strong demand for nitrogen up to 80 kg N per 1000 kg of soybean grain for optimal development and grain productivity (Hungria et al., 2006; Salvagiotti et al., 2008). Soybean can fix N from the atmosphere ranging from 0–450 kg N ha^−1^ (Giller, 2001; Unkovich and Pate, 2000). Under favorable environments for N fixation, over 60 to 70% of the N requirement of the soybean can be derived from BNF (Herridge et al., 2008), while the balance could be derived from the soil N stock. Conversely, Mapfumo (2011) reported that BNF could be as low as 5 kg N ha^−1^ in depleted soils, which are quite common in the smallholder farming systems in sub-Saharan Africa. This would imply reliance on nitrogen fertilizers even for legume crops.

Low soil fertility in SSA is often characterized by low available phosphorous (P), nitrogen (N), organic matter (C_org_), and soil acidity, among others (Sanchez et al., 1997). Such variables must be corrected as they are an integral part of the interaction of legume genotype, rhizobia strain, environment, and crop management which determine the performance of BNF, and legume productivity in general (Gibson et al., 1982; Woomer et al., 2014; Keino et al., 2015; Thuita et al., 2018). Soil organic carbon is a key driver of soil fertility that could even affect the performance of non-limiting factors, when it is below a certain level in a specific soil type (Gruhn et al., 2000). Response to inorganic fertilizers could be enhanced by addition of organic matter (Singh and Ryan, 2015). However, most of agricultural soils in SSA contain low levels of organic carbon due to competing uses of organic residues (Nandwa, 2001; Bationo et al., 2007). Initiatives that promote rhizobia inoculation in legume production in Africa generally recommend application of nutrients such as P. Lately more balanced fertilizer blends have been developed for use with inoculant that do not include N (Streeter and Wong, 1988; Svenning et al., 1996; Woomer et al., 2014; Ronner et al., 2016). This is due to the general assumption that rhizobia would supply the N required by the legume and application in form of mineral N would inhibit nodulation. While such inhibition has been well-documented (Zahran, 1999), this could be different in low fertility soils that are N deficient (Becker et al., 1986). Starter N is sometimes needed to achieve a substantial yield of legumes, including soybean, when the symbiotic N_2_ fixation is unable to provide enough nitrogen (Ohyama et al., 2011).

The objective of the study was thus to assess whether soils with low inoculation response could be improved by application of nitrogen. It was hypothesized that an organic source of nitrogen would perform better than a mineral N fertilizer, given the expected high correlation between organic carbon and total nitrogen in agricultural soils (Rashidi and Seilsepour, 2009).

## 2 Materials and methods

### 2.1 Characterization of the study soils

**Fig. 1:**
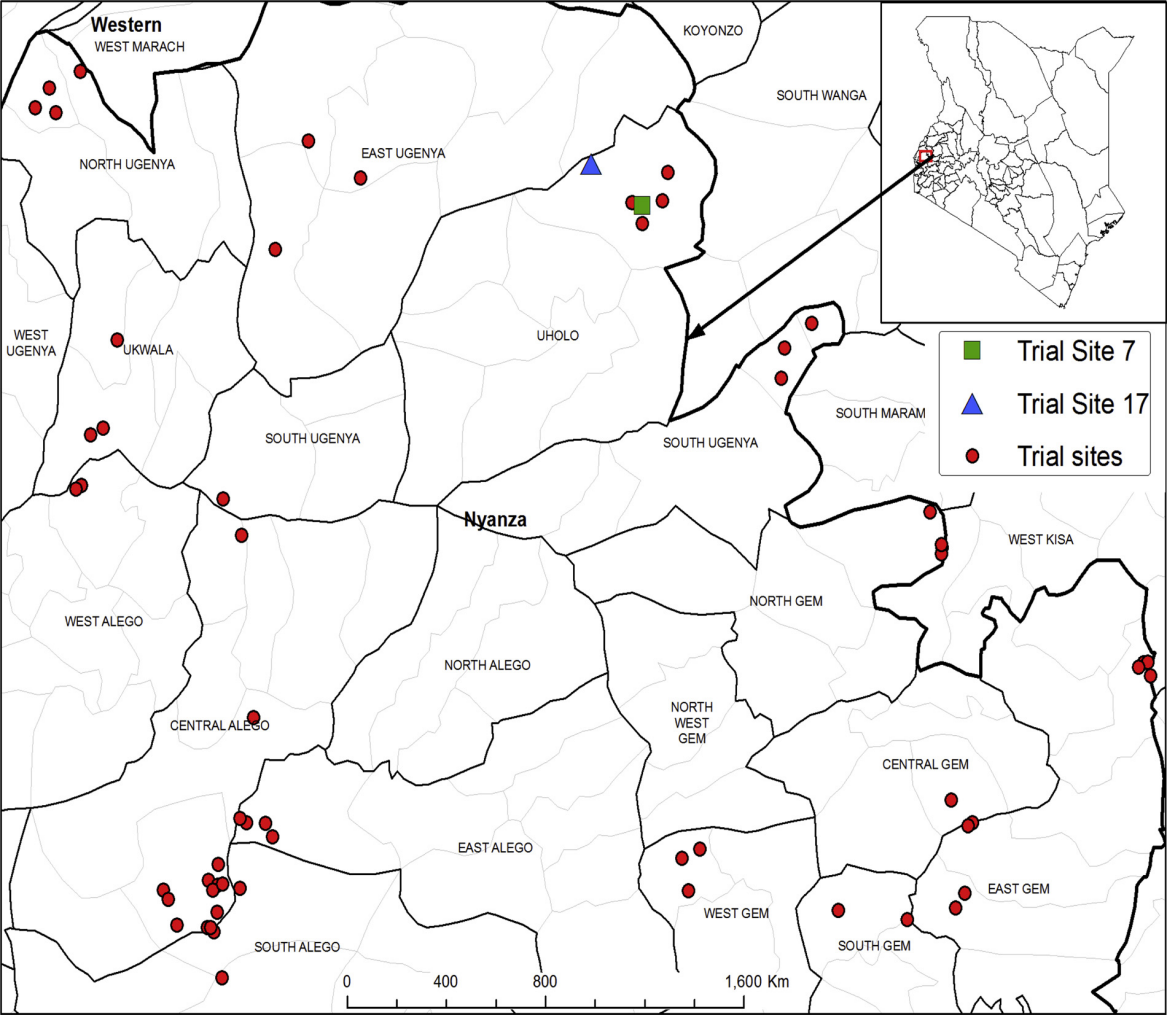
Sites where the soils used in the 1st greenhouse experiment were collected including Trial Site 17 (i.e. Soil B) that was also used in both the 2nd greenhouse experiment and the field trial, and Trial Site 7 (i.e. Soil A) used also in the field trial.

Two greenhouse experiments were established at the International Centre of Insect Physiology and Ecology (ICIPE), Duduville Campus, Nairobi, Kenya. Soils were collected from sixty farms of Siaya County where low soybean response to inoculation was observed (Masso et al., 2016; Thuita et al., 2018) (Fig. 1) and where varied response to an ISFM soybean package had been observed. Soil was collected at a depth of 0–20 cm, air dried under shade and thoroughly mixed to pass through 2 mm sieve. Sub samples were analyzed for physical, chemical and microbiological properties prior to planting. The soils organic Carbon was determined by chromic acid digestion and spectrophotometric analysis (Heanes, 1984), total N (%) determined from a wet acid digest (Buondonno et al., 1995) and N analyzed by colorimetric analysis (Anderson and Ingram, 1993), soil texture was determined using the hydrometer method, soil pH in water determined in a 1:2.5 (w/v) soil:water suspension, available P using the Mehlich-3 procedure (Mehlich, 1984) and the resulting extracts analyzed using the molybdate blue procedure described by Murphy and Riley (1962), exchangeable cations (Ca, Mg, and K) extracted using the Mehlich-3 procedure and determined by atomic absorption spectrophotometry. Estimation of rhizobia in the soils was done using the most probable number count as described by Brockwell et al. (1975); soybean variety TGx1740-2 F was used as a trap crop grown in N free and autoclaved sterile sand. These were grown to flowering stage.

### 2.2 Greenhouse experiments

#### 2.2.1 Greenhouse experiment one

The experiment was laid as Completely Randomized Design (CRD) including: (i) 60 soils collected from the sites indicated in Fig. 1, with N and C_org_ ranges of 0.029-0.21% and 0.53–2.1% respectively, (ii) two treatments uninoculated and inoculated (Legumefix) + fertilizer (Sympal) replicated 3 times for a total number of 360 experimental units. Co-application of Legumefix and Sympal, as an inoculation package, was informed by previous findings of Woomer et al. (2014) and Masso et al. (2016). Sympal is a legume-specific fertilizer blend (i.e. 0N+ 23P_2_O_5_+ 15K_2_O+ 10CaO + 4S + 1MgO + 0.1Zn) and was applied at a rate equivalent to 30 kg P ha^−1^ (assuming 1 ha = 2.24 * 10^6^ kg of soil) and thoroughly mixed with the soil for the inoculated treatments (i.e. ≈300 kg Sympal ha^−1^). Soybean variety (TGx1740-2 F) was selected due to its better nodulation with a range of rhizobia than local varieties used in different parts of Kenya (Wasike et al., 2009). Seeds were surface-sterilized by soaking in 3.5% sodium hypochlorite (NaClO) solution for 2 min and rinsed thoroughly 5 times with sterile distilled water. Soils were weighed to fill in perforated 2.5 kg pots. Legumefix (*Bradyrhizobium japonicum* strain 532c) from Legume technology Inc (UK) was used at a rate of 10 g for 1 kg soybean seeds for the inoculated treatments. Three healthy seeds of uniform size were then planted per pot and thinned to one plant per pot of comparable height and vigor at 2 weeks after planting. Routine management practices such as watering (with distilled water) were carried out until termination of the experiment i.e. at 50% podding. This trial was intended to determine soybean response to co-application of Legumefix and Sympal in various soils characterized by a gradient of nitrogen content.

#### 2.2.2 Greenhouse experiment 2

One of the 60 experimental soils (Trial Site 17 in Fig. 1), that showed low response to inoculation in the first greenhouse experiment based on low nodule fresh weight and shoot dry weight observed was used. The soil N levels were amended either with vermicompost (Phymyx) or urea. Vermicompost was chosen as a slow release form of N compared to urea. A slow N release would reduce the negative effect of N application to nodulation at the early growth stages of soybean. The vermicompost is made by composting for 6 months the substrate used in mushroom production (the substrate is made using wheat straw, sunflower cake, cotton seed cake, chicken manure and then sterilized. Mushroom is grown on it and the substrate is added with 10% minjingu rock phosphate and earthworms and left for 6 months. Sieving is done and then packaged as vermicompost). The soil was collected from an area of 4 by 3 m at a depth of 0–20 cm and homogenized after air drying and sieving. Vermicompost (Vc) was applied at 5 levels with even intervals including a control (i.e. at rates equivalent to 0, 2.5, 5, 7.5 and 10 t Vc ha^−1^). Equivalent amounts of N were applied using urea (46% N). The rates of N were 0, 37, 74, 111, and 148 kg N ha^−1^. Before application vermicompost was analyzed to aid in determining amount of N present to enable application of equivalent amounts from urea fertilizer. The analyses were as follows; total N (0.88%), organic C (7.31%), available P (0.39%), Ca (0.29%), Mg (0.1%), K (0.22%), and pH 6.70. It was also expected to contain trace micronutrients (not determined). Two levels of Legumefix were used as in the first greenhouse experiment. The trial was laid as a CRD and each treatment was replicated 3 times for a total of 60 experimental pots. Planting, management and harvesting were done as described in the first greenhouse experiment. The trial was thus intended to determine whether application of starter N would improve low soybean response to co-application of Legumefix and Sympal in a low fertility soil, and whether there was a systematic difference between vermicompost and urea as sources of N.

#### 2.2.3 Data collection in greenhouse experiments

The plants were harvested at 50% podding. Shoots were cut using a clean, sharp knife at 1 cm above the soil surface. The pots were emptied on a 2-mm sieve and soil washed to isolate the nodules from the roots. Nodule fresh weight, shoot biomass and nodule occupancy were recorded in both greenhouse experiments, whereas in the second greenhouse experiment additional data collected was on nodule effectiveness and N uptake.

### 2.3 Field trial

The field trial was intended to validate the findings of the second greenhouse trials under field conditions with focus on the best performing source of N and determine the yield performance. The trial was conducted at site 17 (Soil B) and site 7 (soil A) (Fig. 1). Site 17 was the farm at which soil was collected for the second greenhouse experiment. Soils from both sites had similar response trends in nodulation and shoot biomass in the first greenhouse experiment even though they did not have the same physical and chemical characteristics (Table 1) and thus were chosen for the field trial validation. The field trial was conducted during the long rains (April to August) 2016 cropping season. The treatments at each site were laid out in a full factorial in randomized complete block design replicated three times. Sympal applied at the rates used in the greenhouse trials (0 and 30 kg P ha^−1^). The five rates of vermicompost used in the second greenhouse experiment was applied i.e. equivalent to 0, 2.5, 5, 7.5, and 10 t ha^−1^, whereas two levels of Legumefix were used i.e. inoculated and uninoculated. The maximum of 10 t ha^−1^ was based on general recommendation for compost application in the region. The plot sizes were 3 m by 3 m and 0.5 m alley between the plots and 1 m between the blocks. Soybean was planted at a spacing of 50 cm (between rows) by 5 cm (within rows) at the onset of long rainy season (April 2016). Vermicompost and Sympal were applied in furrows and mixed with soil before placement of seeds to avoid direct contact with the seed. Seed sterilization and Legumefix application rate were as done in the greenhouse trials. The trials were kept weed free by weeding using hand hoe.

**Table 1:** Selected chemical, microbiological and physical properties of the sixty experimental soils with details of Trial site 7 (Soil A) and Trial site 17 (Soil B).

Variable	Units	Mean	aOverall Minimum	Maximum	SD	CV%	bSoil A	cSoil B
Available P	mg kg^−1^	15.5	1.0	156.6	31.9	205.9	3.1	12.0
pH (H_2_O)	–	5.8	4.3	7.0	0.7	11.5	4.5	5.4
Total N	g kg^−1^	1.1	0.2	2.1	0.4	36.4	0.6	0.8
Organic C	g kg^−1^	13.9	5.3	21	4.3	30.9	8	10.5
Ca	cmol_c_ kg^−1^	5.5	0.4	18.3	3.9	70.8	0.9	5.5
Mg	cmol_c_ kg^−1^	2.3	0.2	8.6	1.7	74.7	0.3	1.5
K	cmol_c_ kg^−1^	0.7	0.1	3.6	0.7	97.2	0.2	0.3
MPN	CFU g^−1^	41.0	0.0	283.0	87.6	208.6	65.0	23.0
Textural class	–	–	–	–	–	–	Clay	Clay

P, phosphorus; K, potassium; Ca, calcium; Mg, magnesium; N, nitrogen; MPN, most probable number; CFU, colony forming units; SD, standard deviation of the means and CV, coefficient of variance.

a Overall stands for mean value across the 60 experimental soils.

b Soil A stands for the mean value for the soil collected from the Trial Site 7.

c Soil B stands for the mean value for the soil collected from the Trial Site 17.

In the field trial, the parameters recorded at 50% podding were nodule fresh weight, nodule effectiveness, nodule occupancy, shoot dry biomass and N uptake, while at harvest grain yield was determined. Eight to ten plants were taken from one of the inner rows about 50 cm from the beginning of the line at 50% podding. Nodules were dug out and washed for nodule fresh weight determination and shoots collected for drying and weighing. A sample i.e. 10% of the total number of nodules counted per treatment was taken and used for determining nodule effectiveness as described by Adjei et al. (2002). At physiological maturity i.e. when 95% of the pods had turned golden yellow, all plants were harvested from the net plot excluding the border rows. Number and weight of all plants were recorded from each plot and grains and haulms separated and weighed. The grains were later oven-dried to a constant weight.

Fresh shoots were dried at 60 °C until constant weight (approximately 48 h) to obtain the dry weights. The shoots were later milled for total N analysis by modified Kjeldahl method. Nitrogen uptake at 50% podding was determined as the product of shoot dry biomass and the respective nitrogen content in the shoot and reported as g N plant^−1^.

### 2.4 Nodule occupancy

Nodule occupancy was then done using Polymerase chain reaction-Restriction Fragment Length Polymorphism (PCR-RFLP) method. This involved amplification and restriction of the 16S–23S rDNA intergenic spacer region. A maximum number of eight nodules from each of the three replicates per treatment (24 nodules) were crushed separately in 150 μl of sterile water and DNA extracted by Krasova-Wade et al. (2003) protocol. Amplification of DNA (PCR) was conducted as described by Navarro et al. (1992) and Ponsonnet and Nesme (1994) using rhizobia specific primers. Due to low number of nodules in the low rates of vermicompost and urea treatments, only the three upper rates and their respective combinations (i.e. 74, 111, and 148 kg N ha^−1^) were examined. In addition, restriction was only conducted for PCR products of a single band of 930–1050 bp using restriction endonucleases *Msp* I. The strains with identical fragment size and number were classified into the same profile and the profiles used to score the inoculant (Legumefix) efficacy in percentages (Thuita et al., 2012).

### 2.5 Data analysis

In the two greenhouse trials and the field trial, analysis of variance (ANOVA) was conducted to assess the effects of the various sources of variation i.e. treatments using SAS version 9.4. The effects of the various factors and their interactions were assessed using standard error of difference (SED) on the mean. The significance level of the models was set at p < 0.05. In the 1^st^ greenhouse experiment, box plots and whiskers were also used to summarize the information on nodule fresh weight and shoot biomass given the large number of experimental soils (i.e. sixty data points). The assessment of nodule occupancy for the greenhouse and field trials was based on PCR-RFLP profiles with similar bp fragments in size after restriction and compared to the IGS profile of strain *B. japonicum* 532c and converted to a percentage of total nodules examined for each IGS profile group.

## 3 Results

### 3.1 Soil properties

Selected properties of the experimental soils including details of Trial Sites 7 and 17 before the beginning of the greenhouse and field trials are presented in Table 1. A wide variation in soil properties was noted with coefficients of variance (CV) ranging from 11 to 208% with most of the parameters falling under what is considered low. There was a strong correlation between total N and C_org_ (r = 0.94). Rhizobia population for the selected soils ranged from 0 to 2.83 × 10^2^ CFU g^−1^ of soil; hence, a response to inoculation was expected.

### 3.2 Nodulation

**Fig. 2:**
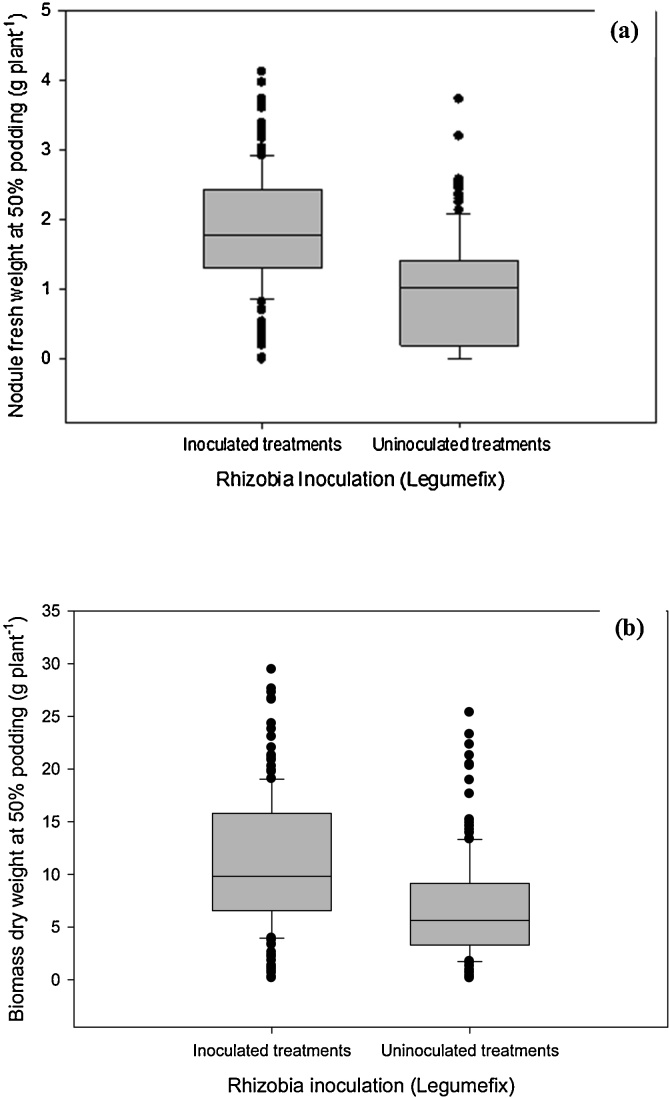
Soybean nodule fresh weight and shoot dry weight in the 1st greenhouse experiment with and without co-application of Legumefix and Sympal (L + S) across sixty soils.

In greenhouse experiment one, the nodule fresh weight (NFW) varied significantly (p < 0.05) across the 60 soils tested irrespective of inoculation. This variation was related to the wide variation in soil properties (Table 2; Fig. 2). On average, inoculation + fertilization treatments had higher NFW than uninoculated soils (no inoculum or fertilizer) (Fig. 2). The NFW was generally low in the soils of poor fertility, which calls for further investigation to raise understanding of the inoculation response variability often observed in field trials.

**Table 2:** Summary of ANOVA for the two greenhouse experiments and the field trial.

Effect	Nodule fresh weight	Nodule effectiveness	Shoot dry weight	Biomass N uptake	Grain yield
First greenhouse experiment					
Soil (S)	***	ND	***	ND	ND
Legumefix (L)+ Sympal (Sy)	***		***		
S × L + Sy	***		***		
second greenhouse experiment					
Amendment^b^ (Amt)	**	**	**	**	ND
L + Sy	***	***	***	***	
Amt × L + Sy	*	*	***	***	
Field trial					
Site (St)	NS	NS	**	NS	NS
Legumefix (L)	***	***	**	***	***
Sympal (Sy)	**	**	**	**	NS
Vermicompost (Vc)	*	***	**	**	*
St × L	***	NS	NS	NS	**
St × Sy	NS	NS	NS	NS	NS
St × Vc	NS	NS	NS	NS	NS
L × Sy	NS	NS	NS	NS	NS
L × Vc	NS	NS	NS	NS	NS
S × Vc	NS	NS	NS	NS	NS
St × L × Sy	NS	*	NS	NS	NS
St × L × Vc	NS	NS	NS	NS	NS
St × Sy × Vc	NS	NS	NS	*	NS
L × Sy × Vc	NS	NS	*	NS	NS
St × L × Vc × Sy	NS	NS	NS	NS	NS

ANOVA: Analysis of variance.

ND: Not determined.

NS: Not significant at p < 0.05; *: p < 0.05; **: p < 0.01; ***: p < 0.001.

aInoculation: Co-application of rhizobia inoculant (i.e. Legumefix) and Sympal (i.e. a legume-specific fertilizer blend).

b Amendment: Application of vermicompost or urea as source of nitrogen.

**Fig. 3:**
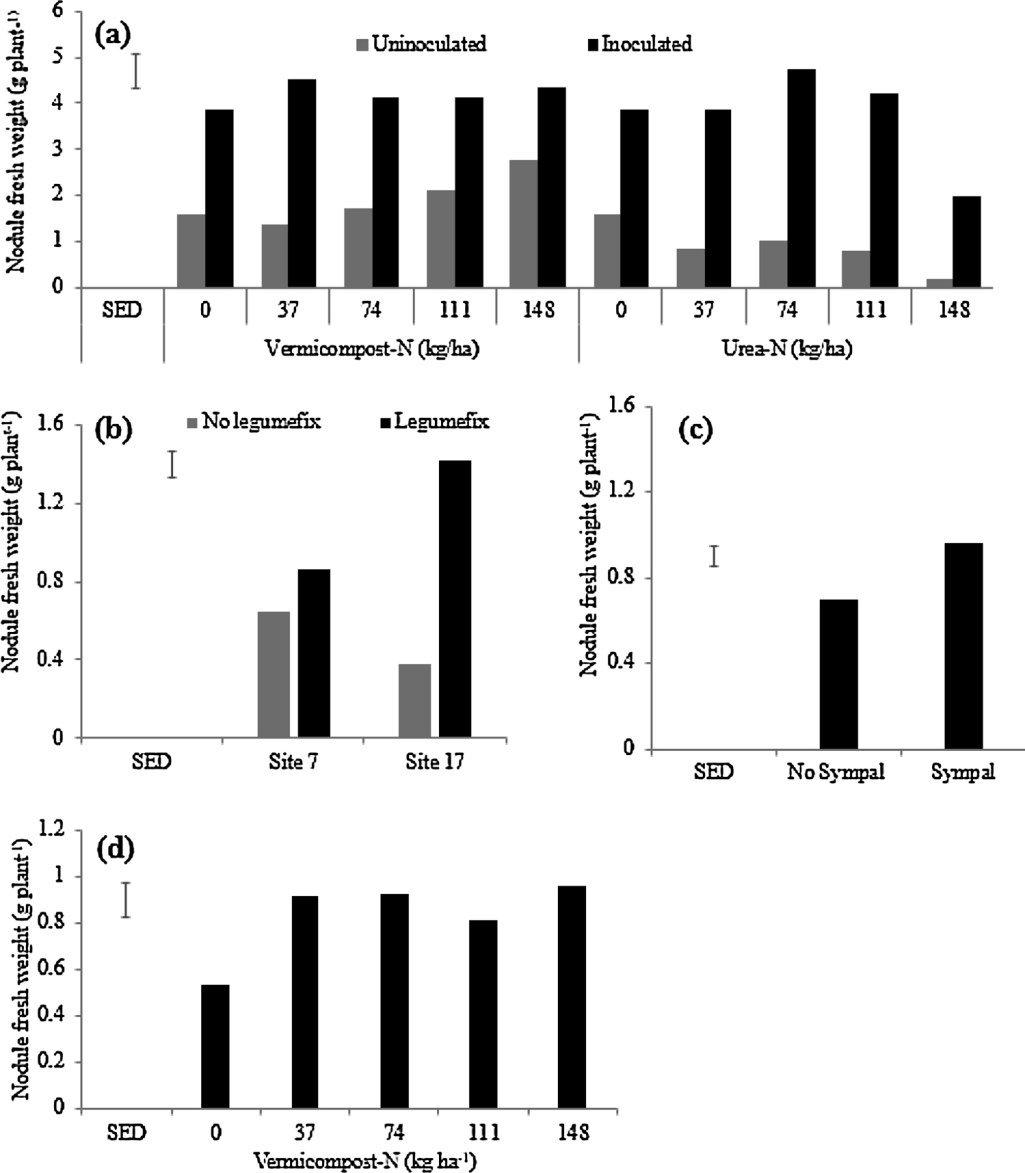
Soybean nodule fresh weight across trials following: (a) N-amendment in the form of vermicompost or urea co-applied with Legumefix and Sympal (L + S) in the 2nd greenhouse trial (Soil B from Trial Site 17); (b) Legumefix application at Trial Site 7 and Trial Site 17 (field conditions); (c) Sympal application in field conditions; and (d) vermicompost in field conditions. The error bars represent the standard error of the difference (SED).

In greenhouse experiment two, co-application of starter N in the form of vermicompost or urea, Legumefix plus Sympal significantly increased nodule fresh weight (p < 0.05) (Table 2). Vermicompost co-applied with Legumefix + Sympal consistently produced a significantly higher nodule fresh weight than urea co-applied with Legumefix and Sympal (Fig. 3a). The higher rates of urea led to a decrease in NFW compared to vermicompost, which may be related to the difference in the availability of N from the two sources.

In the field experiment, NFW was improved by inoculation (P < 0.05) at Trial site 17 compared to Trial site 7 (Fig. 3b), which could be related to the initial fertility level of the sites (Table 1). Conversely, in the absence of inoculation Trial site 7 nodulation was higher (p < 0.05) than Trial site 17 (Fig. 3b), which could be associated with the abundance of soybean nodulating rhizobia at Trial site 7 (Table 1). Regardless of the sites and inoculation, application of Sympal (Fig. 3c) and vermicompost (Fig. 3d) improved NFW, which implied that the nodulation of soybean by native rhizobia could be improved with good soil fertility management.

### 3.3 Nodule effectiveness

**Fig. 4:**
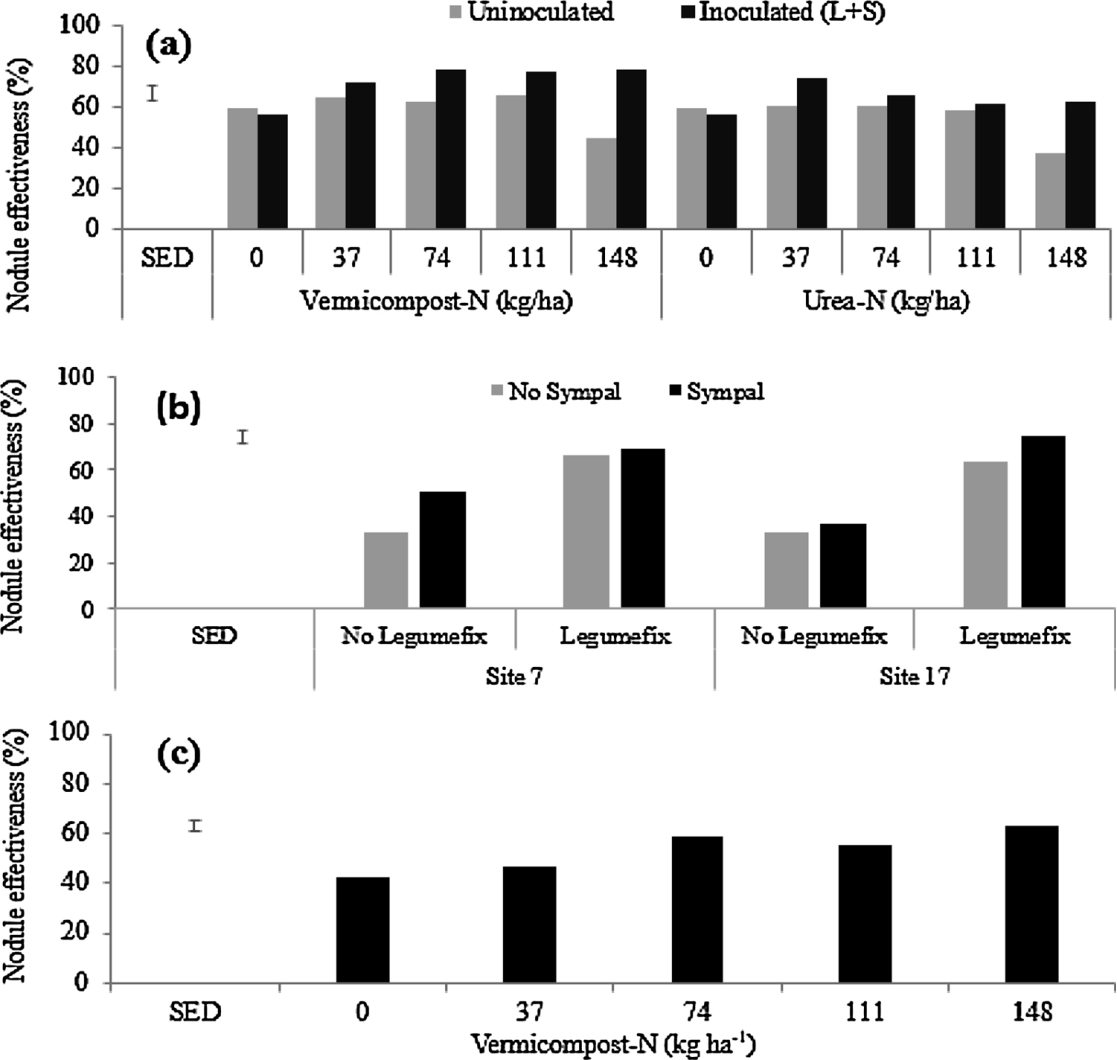
Soybean nodule effectiveness across trials following: (a) N-amendment in the form of vermicompost or urea co-applied with Legumefix and Sympal (L + S) in the 2^nd^ greenhouse trial (Soil B from Trial Site 17); (b) Legumefix and Sympal applications in field conditions at Trial Site 7 and Trial Site 17; and (c) vermicompost application in field conditions irrespective of the sites. The error bars represent the standard error of the difference (SED).

In the greenhouse experiment 2, N-amendment using vermicompost or urea co-applied with Legumefix and Sympal significantly increased the percentage of effective nodules (p < 0.05) (Table 2; Fig. 4a). Vermicompost co-applied with Legumefix and Sympal consistently had higher percentage of effective nodules as compared to urea co-applied with Legumefix and Sympal (Fig. 4a).

In field conditions, inoculation at Trial Site 7 and Trial Site 17 improved nodule effectiveness at both sites, but co-application with Sympal showed better performance at Site 17 than Site 7 when compared to inoculation without Sympal (Fig. 4b). Conversely, in the absence of inoculation, Sympal improved nodule effectiveness at Site 7 than Site 17, while when Sympal was not applied nodule effectiveness was similar at both sites (Fig. 4b). While Sympal contributed to the improvement of the nodule effectiveness, the magnitude of the response demonstrated that inoculation was very critical to enhance the percentage of effective nodules. There was a significant increase of nodule effectiveness (p < 0.05) following vermicompost application. The highest nodule effectiveness was found at total N rate ≥ 74 kg ha^−1^ irrespective of inoculation and Sympal at both sites (Fig. 4c).

### 3.4 Nodule occupancy

In the greenhouse experiment 1, three IGS profile groups were obtained from PCR-RFLP analysis. The IGS profile I (inoculant strain) was dominant in the inoculated soils, while IGS profile II and III (indigenous strains) were dominant in the non-inoculated soils (Table 3). In the greenhouse experiment 2, nodule occupancy by the inoculant strain consistently increased with increase in vermicompost rates in the uninoculated treatment, showing that the strain in the rhizobia inoculant is present in the study region due to previous history of soybean cultivation with the inoculant strain in the two sites (Table 4). Increased rate of N from vermicompost up to 148 kg ha^−1^ did not suppress nodule occupancy by the inoculant strain. At the rate 148 kg N ha^−1^ from urea nodulation was suppressed to the extent no nodules were found irrespective of inoculation. This was attributed to the slow release of N from not inhibiting nodulation while the high rates of N from urea did inhibit nodulation. For the rates of 74 and 111 kg N ha^−1^, under co-application of the rhizobia inoculant and Sympal, all the nodule analyzed were occupied by the inoculant strain. Based on the results reported in Fig. 3a (nodule fresh weight) and Fig. 4a (nodule effectiveness) at 148 kg N ha^−1^ from urea, there was a likelihood some native strains that can nodulate soybean were not detected by the specific primers used to assess the nodule occupancy and thus total number of nodules analyzed were not equal in all the treatments. This often occurs when some bacteria have acquired genes that enable nodulation but may not have all the required genes to allow detection by the set of primers; further investigation would be required.

**Table 3:** Summary of nodule occupancy analysis for the greenhouse experiments as affected by inoculation and vermicompost or urea as source of starter N.

Greenhouse experiments					
N-Amendment	Treatment	Nodules analyzed	Nodule occupancy by IGS groups (%)		
			I^a^	II^b^	III^b^
First greenhouse experiment					
NA	With L + Sy	184	91	2	7
NA	Without L + Sy	75	46	13	40
Second greenhouse experiment					
Vermicompost	74 N + Sy^c^ + L	12	100	0	0
	111 N + Sy + L	13	100	0	0
	148 N + Sy + L	12	100	0	0
	74 N	5	60	40	0
	111 N	8	63	37	0
	148 N	13	77	23	0
Urea	74 N + Sy^c^ + L	14	100	0	0
	111 N + Sy + L	12	100	0	0
	148 N + Sy + L	12	83	17	0
	74 N	7	100	0	0
	111 N	6	67	33	0
	148 N	0	0	0	0

N: Nitrogen; L: Legumefix; Sy: Sympal; 74, 111, and 148 stands for kg N ha^−1.^

a IGS group I: For the strain in Legumefix.

b IGS group II and III: For indigenous rhizobia strains.

c Sympal was applied at a rate of 300 kg ha^−1^ supplying P, K, Ca, S, Mg, and Zn at rates of 30, 38, 21, 12, 1.8, and 0.24 kg ha^−1^ respectively.

**Table 4:** Summary of nodule occupancy analysis for the field trial as affected by vermicompost as source of starter N and inoculation.

Field trial					
Trial Site	Treatment	Nodules analysed	Nodule occupancy by IGS groups (%)		
			I^a^	II^b^	III^b^
7 (Soil A)	74 N + Sy + L	18	94	0	6
	111 N + Sy + L	18	94	0	6
	148 N + Sy + L	14	79	0	21
	74 N + Sy	2	0	0	100
	111 N + Sy	1	0	100	0
	148 N + Sy	1	0	0	100
	74 N + L	2	50	0	50
	111 N + L	0	0	0	0
	148 N + L	4	50	0	50
17 (Soil B)	74 N + Sy + L	14	100	0	0
	111 N + Sy + L	20	85	0	15
	148 N + Sy + L	20	85	0	15
	74 N + Sy	4	0	50	50
	111 N + Sy	1	0	100	0
	148 N + Sy	4	0	25	75
	74 N + L	6	100	0	0
	111 N + L	7	0	0	100
	148 N + L	5	100	0	0

N: Nitrogen from Vermicompost; L: Legumefix; Sy: Sympal; 74, 111, and 148 are the rates of N (kg ha^−1^). Sympal was applied at a rate of 300 kg ha^−1^ supplying P, K, Ca, S, Mg, and Zn at rates of 30, 38, 21, 12, 1.8, and 0.24 kg ha^−1^ respectively.

a IGS group I: For the strain in Legumefix.

b IGS group II and III: For indigenous rhizobia strains.

In the field trial, highest inoculant strain recovery (nodule occupancy) was observed with the combination of Legumefix, vermicompost and Sympal demonstrating the relevance of the combination to supply additional nutrient, organic matter, and rhizobia particularly in the low fertility soil at Site 7 (Table 4). Highest inoculant strain recovery was attained when vermicompost was applied at 74 kg N ha^−1^ and 111 kg N ha^−1^ and combined with Sympal and Legumefix at Site 7 (94%), while there was a slight reduction at 148 kg N ha^−1^ for the same inputs though the inoculant strain recovery was still higher than 66% (Table 4). At Site 17, which was slightly more fertile than Site 7, the value addition of co-applying Legumefix and Sympal in the presence of vermicompost was reduced, except at 111 kg N ha^−1^ (Table 4). In the absence of the rhizobia inoculant, co-application of vermicompost and Sympal did enhance the nodule occupancy by native strains, although they were less effective based on the results on nodule effectiveness (Fig. 4b). In general, a consistently higher percentage of nodules occupied by the inoculant strain was observed in the inoculated and amended soils for both greenhouse and field conditions at moderate levels of N (i.e. 74 and 111 kg N ha^−1^ regardless of the source of N). This suggests that the introduced strain was more competitive in the amended soils and explains the higher percentage of effective nodules (Fig. 4b). The recovery of the inoculant strain from the uninoculated treatments (especially in the second greenhouse experiment) was attributed to previous history of soybean cultivation with the same inoculant on the two farms.

### 3.5 Shoot biomass

**Fig. 5:**
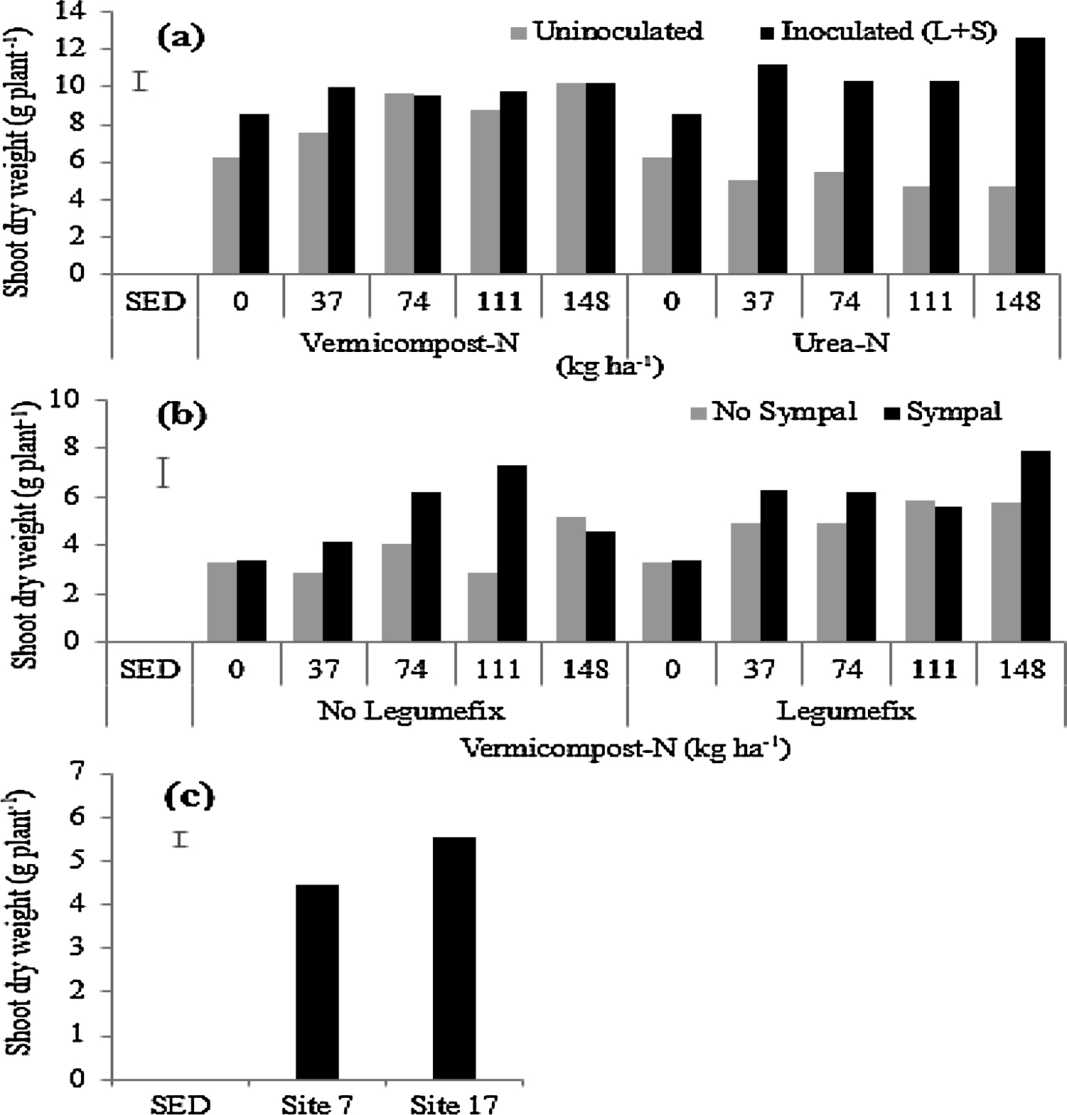
Shoot dry weight across trials following: (a) N-amendment in the form of vermicompost or urea co-applied with Legumefix and Sympal (L + S) in the 2nd greenhouse trial (Soil B from Trial Site 17); (b) various combinations of vermicompost, Legumefix, and Sympal in field conditions; and (c) locations. The error bars represent the standard error of the difference (SED).

On average, the inoculated treatment gave a higher shoot dry weight than the uninoculated soils in the first greenhouse trial, with an increase of 38% over the control (Fig. 2), but the improvement of shoot biomass following inoculation significantly varied across soils (Table 2). In the second greenhouse experiment, co-application of N amendments (vermicompost or urea) with Legumefix and Sympal enhanced shoot dry biomass (Fig. 5a). When N was applied as vermicompost, the value addition of Legumefix and Sympal was found at the low rate of vermicompost (equivalent 0 and 37 kg N ha^−1^). Conversely, when N was applied as urea, there were significant differences in shoot biomass between Legumefix + Sympal + urea compared to urea alone in all the urea rates. The difference between the two sources of N can be related to the additional nutrients in vermicompost compared to urea that only supplied N. Across treatments, the highest shoot dry biomass at 50% podding was found at 148 N kg^−1^ applied as urea and combined with Legumefix and Sympal. This could be attributed to the fact that nitrogen from urea was readily available for uptake and resulted in vigorous vegetative growth and more biomass accumulation at the early stage of the crop with minimal N losses in the greenhouse conditions.

In the field conditions, co-application of vermicompost and Legumefix significantly improved shoot dry biomass compared to vermicompost in the absence of Legumefix, particularly when Sympal was not applied (Fig. 5b). When Sympal was added to both combinations (i.e. vermicompost with and without Legumefix), the difference in shoot dry biomass was reduced, which could be related to improved utilization of N. On average, the shoot dry biomass was higher at Site 17 than Site 7 irrespective of the treatments (Fig. 5c), which was consistent with the initial chemical properties of the two sites (Table 1).

### 3.6 Shoot biomass N uptake

**Fig. 6:**
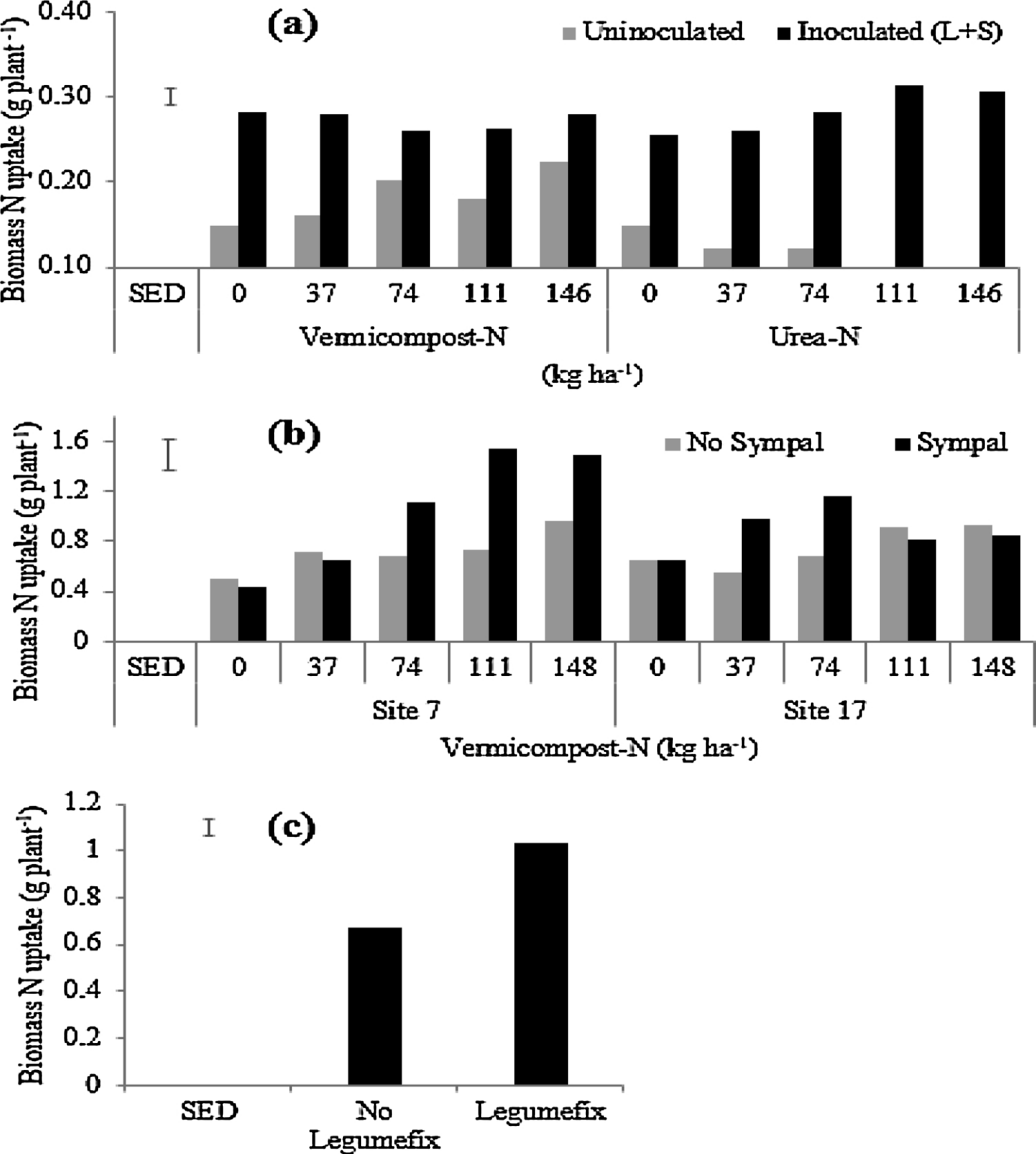
Biomass N uptake across trials following: (a) N-amendment in the form of vermicompost or urea co-applied with Legumefix and Sympal (L + S) in the 2nd greenhouse trial (Soil B from Trial Site 17); (b) co-application of vermicompost and Sympal at two sites; and (c) rhizobia inoculation in field conditions. The error bars represent the standard error of the difference (SED).

In the second greenhouse experiment, co-application of vermicompost or urea as source of starter N with Legumefix and Sympal significantly increased biomass N uptake when compared to the starter N sources in the absence of Legumefix and Sympal (Table 2; Fig. 6a). When vermicompost or urea was not co-applied with Legumefix and Sympal, increased rates of vermicompost enhanced biomass N uptake, while increased rates of urea reduced N uptake. This could be related to the improved soybean growth in the presence of vermicompost related to the additional nutrients in the inputs, which would have improved the root system development (data not collected) and consequently N uptake. Application of Legumefix and Sympal to both starter N treatments further enhanced plant development and therefore N uptake.

In the field conditions, co-application of vermicompost and Sympal enhanced N uptake at Site 7 (which was less fertile) more than Site 17 (Fig. 6b). In the absence of Sympal, N uptake was similar at both sites when vermicompost was applied. On average, rhizobia inoculation showed higher N uptake than uninoculated plants, irrespective of trial sites, vermicompost and Sympal (Fig. 6c).

### 3.7 Grain yield

**Fig. 7:**
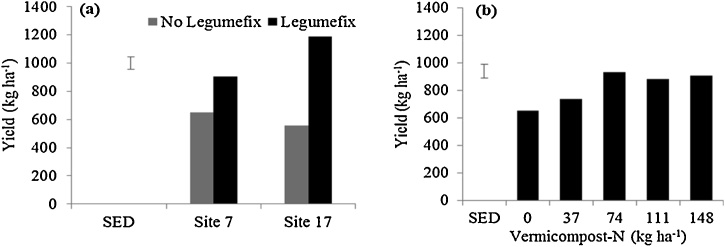
Soybean grain yield in field conditions following: (a) rhizobia inoculation at two sites; and (b) vermicompost application. The error bars represent the standard error of the difference (SED).

When soybean was inoculated, yields were higher at Site 17 than Site 7, while both sites had similar yields in the absence of inoculation (Fig. 7a). Hence, the apparent difference in soil fertility at the two sites (Table 1), was not enough to show a difference in yields without soil amendment. Amendment with vermicompost increased soybean grain yield from N rate of 74 kg ha^−1^ compared to the absolute control (Fig. 7b); this rate of N was equivalent to five metric tons of vermicompost ha^−1^. Grain yields significantly increased on amendment (475, 709, 856, 880, 966 kg ha^−1^) after application of vermicompost at 0, 37, 74, 111, and 148 kg N ha^−1^ respectively. All the measured parameters reported correlated significantly to grain yields particularly at Site 7 (data not shown), which showed that amending low fertility soils using various combinations of inputs like rhizobia inoculant, Sympal, and vermicompost could enhance soybean growth and yield assuming no other limiting factors.

## 4 Discussion

In this study, the overall effects of four key factors i.e. site (soil), rhizobia inoculant, starter N (vermicompost or urea), and a legume-specific fertilizer blend (Sympal) and their interactions on soybean productivity traits including nodulation, nodule effectiveness, nodule occupancy, shoot dry weight, N uptake, and yield were evaluated. These productivity traits were improved by various combinations of the three inputs, but in most of the cases there was a significant site or soil effect. Previous studies demonstrated that legume response to inoculation is generally affected by (i) legume genotype, (ii) rhizobia strain, (iii) environmental factors like soil fertility, soil amendments, and water management, and (iv) crop management such as weeding, spacing, pest and disease control (Giller et al., 2013; Woomer et al., 2014;) as well as the nature of existing soil population of rhizobia (Yang et al., 2018). In this study, the focus was on aspects related to soil fertility improvement to enhance soybean productivity traits. The hypothesis that starter N, particularly in organic form, would improve soybean response to rhizobia inoculants and legume specific fertilizer blends (without N) in low fertility soils was confirmed and it is crucial to understanding the underlying mechanisms.

### 4.1 Need for starter N to improve soybean response to inoculation in low fertility soils

The soils used in the three experiments were low in nitrogen levels as reported by findings of Masso et al. (2016). Nitrogen is a major limiting factor in plant growth and development. In low fertility soils, there is a need to explore various nutrient replenishment avenues to establish best practice management options for improved soybean response to inoculation (Woomer et al., 2014). In soils with low nitrogen, a moderate amount of ‘starter nitrogen’ is required by the legume plant for nodule development, root and shoot growth before the onset of BNF (Herridge et al., 1984; Goi et al., 1993). In the low N soil used in the second greenhouse experiment, amendment with two nitrogen sources (i.e. vermicompost and urea) significantly increased soybean productivity traits suggesting the nitrogen supplied played a role in soybean growth before a symbiotic relationship of the host crop and rhizobia was fully functional. Although insignificant responses of soybean to starter N have been reported (Mendes et al., 2003), positive responses have been reported by several studies (Lamb et al., 1990; Starling et al., 1998; Osborne and Riedell, 2006; Sohrabi et al., 2012; Janagard and Ebadi-Segherloo, 2016) which demonstrates the need of starter N, particularly in low fertility soils as it was the case in this study. There is still a need to determine the threshold value of soil N content (% or g N kg^−1^ soil) above which, starter N would not be required.

### 4.2 Preference for an organic source of starter N in low fertility soils

The vermicompost treatments performed better in all the measured variables compared to the urea treatments. Although N supplied by urea was readily available for the plant uptake, N alone could not explain the significant increase in the soybean growth traits observed. Vermicompost not only was a source of slow-released N, but also other essential nutrients such as Ca, Mg K among others, which are essential for optimal plant growth. Organic sources of N also improve soil organic carbon, which has a significant effect on soil fertility including rhizobial survival (Swanepoel et al., 2011). In general, soil total N and organic matter are highly correlated as found in this study. In low organic matter soils, organic amendments act as source of nutrients, improve soil structure, increase biodiversity and activity of the microbial population (Albiach et al., 2000; Liu et al., 2008). Use of organic amendments to improve nutrient-depleted soils of SSA regions in general and western Kenyan in particular (Woomer et al., 2014) would improve physical, chemical and biological characteristics of soil (Albiach et al., 2000). This implies that soil amendment with vermicompost, or similar organic inputs, would be a good practice to improve soybean response to inoculation as nodulation and nodule effectiveness were not suppressed up to a rate of 148 kg vermicompost-N ha^−1^. Furthermore, use of organic amendments including organic fertilizers in integrated soil fertility management to supply both nutrients and organic matter would be more conducive to sustainability and resilience of the cropping systems than sole application of inorganic fertilizers.

### 4.3 Balanced fertilization to improve soybean response to inoculation

Significant variation of soybean response to rhizobial inoculation was observed across the sixty soils under greenhouse conditions, which was confirmed under field conditions at two sites. Success of soybean rhizobia inoculation depends on soil fertility and site location (Date, 2000). Based on recommendations by Tadesse (1991) and the soil analysis results, the study soils from sixty locations in western Kenya had very low to moderate fertility, which was in agreement with the report of Woomer et al. (2014). This wide variation in soil properties with most of the variables falling under low to very low (Okalebo et al., 2002; FAO, 2006) could explain the variation of the soybean response to inoculation. Similar findings of spatial variation of soybean response to rhizobia inoculants across locations was reported by Masso et al. (2016). Edaphic factors such as nutrient P and N availability and soil pH determine the effectiveness of inoculant used (Masso et al., 2015; Yang et al., 2018). This has also been confirmed in our ongoing investigation on the effect of soil acidity and liming on soybean productivity traits under inoculation (unpublished). Soil amendment to improve the fertility including balanced fertilization is therefore crucial to reduce the spatial variability of soybean response to inoculation, assuming no other limiting factors.

Under the field conditions, nodule fresh weight and effectiveness were improved by the application of Sympal and/or vermicompost, shoot dry weight was enhanced by co-application of vermicompost, Sympal, and Legumefix, while combination of vermicompost and Sympal increased biomass N uptake and vermicompost boosted grain yield. This was in line with the findings of Mpepereki et al. (2000); Adeli et al. (2005); Mishra et al. (2010); Tahir et al. (2009), and Devi et al. (2013). Soil amendment improved the effectiveness of the nodules and the competitiveness of the introduced strain to occupy a significant number of nodules, as shown by the nodule occupancy. Vermicompost and Sympal contained various nutrients including macro-, secondary and micro- nutrients, which are essential to plant growth and effective nodulation. A package of fertilization interventions based on proper soil fertility diagnosis in legume cropping systems including among others organic inputs, legume-specific fertilizer blend conducive to nodule formation, and rhizobia inoculants found efficacious would be more effective than sole application of a single component of the package (O’Hara et al., 2002; Zhang et al., 2002; Rathke et al., 2005; Sarr et al., 2005; Tahir et al., 2009); though profitability analysis would be required to inform the package choice to recommend. Hence, current development initiatives that promote rhizobia inoculation without necessary soil fertility diagnosis or only focus on co-application of phosphorus and rhizobia inoculants only must be revisited to consider balanced fertilization. Effective legume rhizobia inoculation only adds N to the cropping systems and there is therefore a need to ensure that the other nutrients are available at appropriate levels for optimum plant growth. Availability of essential nutrients as reported by Schulze (2006); Divito and Sadras (2014) and Yoseph and Worku (2014) and moderate levels of nitrogen generally enhance nodule formation and functioning. High rates of nitrogen fertilizers however have been shown to inhibit nodule formation in both controlled and field conditions (Otieno et al., 2007; Ohyama et al., 2011; Bekere et al., 2013; Keino et al., 2015). Hence, investigations to determine the threshold values, depending among others on soil types, below which starter N would be required to improve legume response to inoculation in low fertility soils, are needed.

### 4.4 Effectiveness of inoculant rhizobial strains

Response to rhizobia inoculation is expected in soils containing few rhizobia or where the compatible rhizobia of the host legume are absent (Abaidoo et al., 2007; Fening and Danso, 2002). The rhizobia populations in the sixty soils were below 1.0 × 10^3^ CFU g^−1^ of soil, which has been reported as the minimal population of native rhizobia for a response to inoculation to be achieved for legume crops like soybean (Thies et al., 1991). The capacity of an inoculant strain to occupy nodules on the host depends on environmental factors such as presence of soil rhizobia and soil type (Thies et al., 1992; Vlassak et al., 1997). The increased nodule weight and shoot biomass over the control due to rhizobia inoculation indicated that the introduced strain was more effective than the indigenous bradyrhizobia that have become established in the soils tested. This was in line with studies of Rahmani and Saleh-Rastin (2001); Bai et al., 2002Bai et al. (2002); Janagard and Ebadi-Segherloo (2016) and Hungria and Mendes (2015) who reported significant increases in nodulation and biomass with rhizobia inoculation. The soybean increased biomass, nodulation and effective nodules due to inoculation confirms the need to inoculate soybean seeds in the soils of the selected sites. Even though the variety TGx1740-2 F is promiscuous, nodule occupancy analysis confirmed successful inoculation. Inoculation with Legumefix significantly increased percentage of effective nodules and nodule occupancy both in greenhouse and field experiment. Nodule effectiveness and occupancy are important indicators of efficient soybean rhizobia symbiosis (Vlassak et al., 1997; Adjei et al., 2002). The yield increase following application of Legumefix at both sites was in line with findings of Egamberdiyeva et al. (2004); Tahir et al. (2009); Hussain et al. (2011) and Ronner et al. (2016). As mentioned above, to optimize soybean response to rhizobia inoculants like Legumefix, soil amendment with organic sources of nutrients and legume-specific fertilizer blend in low fertile soils will be of great importance not only in the Siaya County of Kenya, but also across sub-Sahara Africa where nutrient depletion is widely spread (Liu et al., 2010; Vanlauwe et al., 2015). In addition, there is need to address issues related to factors like legume genotype, efficacy of rhizobia strains, as well as good crop and water management among others.

## 5 Conclusion

Soil amendment with vermicompost, Legumefix, and Sympal in low fertility soils increased soybeans productivity traits including yields. Soybean response to inoculation was affected by the soil properties. Vermicompost supplied both nutrients and organic carbon, while Sympal contributed additional nutrients, which improved the nutrient status of the low fertility soils and consequently soybean response to inoculation. To fully benefit from BNF development initiatives with legume inoculation components, there is need to consider soil fertility status of the target areas. The technology must seeks to overcome the possible limiting factors to the genetic potential of the legume and rhizobia to be used to fix N. Starter N in the form of vermicompost in low fertility soils at the rates used in this study did not suppress soybean nodulation, and it improved the productivity traits of the crop. However, further investigation is required to determine the threshold value of soil N concentration above which starter N is not recommended when rhizobia inoculants are applied.
